# The Influence of Berry-Derived Polyphenol Supplementation on Exercise-Induced Oxidative Stress and Cardiovascular Health in Physically Active Individuals

**DOI:** 10.3390/antiox13121561

**Published:** 2024-12-19

**Authors:** Joanna Ruszkowska, Wojciech Drygas, Magdalena Kwaśniewska

**Affiliations:** 1 Department of Social and Preventive Medicine, Medical University of Lodz, 90-752 Lodz, Poland; wojciech.drygas@umed.lodz.pl (W.D.); magdalena.kwasniewska@umed.lodz.pl (M.K.); 2 World Institute of Family Health, The President Stanisław Wojciechowski Calisia University, 62-800 Kalisz, Poland

**Keywords:** berry fruit, oxidative stress, physical activity, sport, polyphenols, training, cardiovascular risk

## Abstract

Numerous studies have documented that high-intensity or prolonged exercise is associated with increased oxidative stress and modification of antioxidant status. Polyphenol-rich dietary supplements seem to be the compounds that can upregulate the endogenous antioxidant defense system and consequently prevent muscle damage, support recovery. As berry fruits are at the top of the list of the richest polyphenol food sources, supplements containing berries have become the subject of interest in the context of counteracting exercise-induced oxidative stress and the development of cardiovascular diseases. The purpose of this review is to summarize current knowledge on the effects of berry-derived polyphenol supplementation on exercise-induced oxidative stress and cardiovascular health in physically active individuals. Based on the available literature, blackcurrant supplementation, with its richest version being New Zealand blackcurrant extract, is the most commonly explored berry fruit, followed by chokeberries and blueberries. Although several studies have documented the significant and beneficial influence of berry-derived supplements on redox status and cardiovascular response, some inconsistencies remain. The presented findings should be interpreted with caution due the limited number of available studies, particularly with the participation of physically active individuals. Further research is needed to reveal more comprehensive and accurate data concerning the impact of berry-derived supplements on exercise-induced outcomes taking into account the type of supplement, time of administration, and dosage.

## 1. Introduction

The beneficial effect of some specific substances of plant origin on human health have been known since ancient times [1,2]. A robust body of evidence underscores the protective role of adequate nutrition in terms of the incidence and mortality associated with chronic diseases, quality of life, and longevity [3,4,5]. Several leading health organizations advocate for well-balanced diets rich in highly potent nutrients as a means of achieving substantial health benefits [6,7,8,9,10,11].

Among the numerous elements of nutrition, antioxidants are one of the most potent compounds in relation to health promotion and chronic disease prevention. It is well documented that maintaining the balance between free radicals and antioxidants is fundamental in both prevention and treatment [12,13,14]. Antioxidants are known for their various properties delaying or preventing the oxidation of other chemicals. They improve the in vivo protection against reactive oxygen species (ROS) involved in pathogenesis of several health disorders like atherosclerosis, diabetes, cancers, neurodegenerative diseases, etc. [15,16,17]. Apart from a few endogenous antioxidants that are naturally produced in the human body, the majority of important antioxidants are supplied by the diet (exogenous antioxidants). Among the exogenous antioxidants, four main groups of compounds can be distinguished: vitamins, carotenoids, trace elements, and polyphenols [18]. The links between antioxidant vitamins, carotenoids, and microelements, and the risk of cancer or cardiovascular disease (CVD) have already been broadly discussed in the literature [19,20,21,22,23,24,25,26,27].

The latest scientific studies focus on the influence of dietary polyphenols on health outcomes. There is growing evidence that the intake of polyphenols (dietary or as supplements) may modify the risk of chronic diseases in different sociodemographic groups and health conditions.

Polyphenols are present in a variety of foods and beverages of plant origin. In order to identify the richest dietary sources of polyphenols, Pérez-Jiménez (2010) used the Phenol-Explorer database, which allowed the retrieval of information based on the content of 502 polyphenol glycosides, esters, and aglycones in 452 foods [28]. According to the list of the 100 richest dietary sources of polyphenols, the top 20 food items are mainly fruits. Among these fruits, there are several berries including the black chokeberry, the black elderberry, lowbush and highbush blueberries, as well as the blackcurrant. Importantly, when analyzed by polyphenol concentration in a serving, the abovementioned berries are placed at the very top of the ranking [28].

Due to the biological potential of polyphenols, they are also the subject of research in the context of physical activity (PA), exercise efficiency, and physical performance among physically active persons and competitive athletes [29,30,31].

It has been documented that high-intensity endurance exercise as well as prolonged training requiring significant muscle power may induce excessive oxidative stress and acute inflammation, even resulting in muscle damage [32,33,34]. The human body produces several protective antioxidant mechanism and enzymes like superoxide dismutase (SOD), glutathione peroxidase (GPx), catalase (CT), glutathione reductase (GR), etc. [35]. However, they may not be effective enough to neutralize the harmful effects of excessive exercise, prolonged training, or overloaded exertion [36,37].

Due to the well-known antioxidant benefits of dietary polyphenols, investigating physiological interactions between exercise and polyphenol supplements is not a new concept. However, despite the increasing amount of research, there are several inconsistencies concerning the direct effects of polyphenol supplementation in recreational, competitive, or elite athletes. As berry-derived polyphenols are highly potent antioxidant compounds, studies focused on assessing their properties in relation to exercise are particularly interesting. Of note, many of the studies were performed using a mixture of active substances, so drawing conclusions about the direct role of berry-derived polyphenols seem difficult.

The purpose of this review is to summarize the current knowledge on the effects of berry-derived polyphenol supplementation on exercise-induced oxidative stress and cardiovascular health in physically active individuals.

The present review highlights the potential properties of selected berry-derived polyphenols used alone, without any additional bioactive compounds, in order to more directly explore their properties.

## 2. Exercise and Oxidative Stress

Physiologically, ROS are mainly generated during muscle contraction as byproducts of cellular respiration released by mitochondria. They can be beneficially utilized by immune cells to destroy viruses and bacteria. In order to avoid the toxic effects of aerobic metabolism, organisms (including humans) have developed a defense system to maintain oxidative–antioxidative balance, which includes enzymes (SOD, GPx, CT, GR), blood plasma proteins (ceruloplasmin, transferrin, ferritin, and albumin), and exogenous factors (dietary antioxidants). The excessive production of ROS and/or insufficient antioxidant defenses lead to oxidative stress.

Regular moderate physical activity has been shown to enhance the antioxidant defense system by improving the activity of endogenous antioxidant enzymes [38,39]. The phenomenon of exercise-induced oxidative stress was first observed over 40 years ago [40,41]. Although a large body of evidence confirming the beneficial effects of regular moderate PA was gathered, there is no consistency regarding the impact of intensive or prolonged training. It has been documented that exhaustive exercise may have adverse effects, mainly through the mechanism of generating excessive ROS [42,43,44,45]. Immense ROS production results in the oxidation of polyunsaturated fatty acids of membrane lipids. In turn, lipid peroxidation of muscle cell membranes entails a decrease in membrane fluidity, and an increase in their non-specific permeability, resulting in changes in membrane functionality and muscle cell damage. The consequence of these changes are observed as the development of inflammation, delayed muscle soreness, and elevated levels of intramuscular enzymes, such as creatine kinase (CK). Exercise-induced oxidative stress can contribute to muscle fatigue and a decrease in exercise capacity [46]. Free-radical damage can also affect proteins enzymes as antioxidant enzyme activity decreases due to system overload [14,47,48].

The antioxidant capacity is usually measured by SOD and/or GPx activity, and the plasma level of malondialdehyde (MDA), a marker of lipid peroxidation, which is an indicator of oxidative stress. In turn, the degree of muscle damage is commonly evidenced by an increase in the plasma CK or lactate dehydrogenase activity. Importantly, CK activity can remain elevated even 4 days after intensive exercise [43].

Several studies have documented the dynamic increase in ROS generation and oxidative stress after a workout as well as the rising activity of antioxidant processes as a counteraction to radical production. Various models of intensive exercises (for example, repeated sprints, ultra-endurance marathon running, etc.) may result in changes in numerous plasma oxidant/antioxidant biomarkers [49,50]. However, some studies revealed that markers of exercise-related oxidative/antioxidant status could remain unchanged [51,52], suggesting a probable adaptation process to a regular high level of physical activity. Importantly, redox reactions may depend on several other factors like the type of exercise, the sport season, the training load, etc. [53].

Concluding, it should be underlined that physical activity has a well-documented influence on redox status. However, in spite of growing evidence for the beneficial effects of moderate PA level, several inconsistencies remain regarding excessive and prolonged exercises. The majority of studies showed an increased risk of excessive oxidant activity and insufficiency of the antioxidant system in highly active individuals. Therefore, there is an interest in searching for effective methods aimed at reducing the detrimental effects of oxidative stress.

## 3. Selected Berry-Derived Polyphenol Supplementation and Oxidative Stress in Physically Active Individuals

Polyphenols are bioactive plant secondary metabolites engaged in many vital processes such as growth, pollination, and resistance to pathogens and the environment [54]. When supplied with the diet, they have the ability to react with other compounds in the human body in order to prevent excessive oxidative stress. From a chemical point of view, polyphenols are compounds with two or more hydroxyl groups attached to at least one benzene ring, and they can be classified as flavonoids (anthocyanins, flavanols, flavanones, flavonols, flavonones, and isoflavones) and non-flavonoid polyphenols (lignans, phenolic acids, stilbenes, xanthones, and tannins) [24,55]. Due to their antioxidant properties, polyphenols have become extensively examined in studies including experiments on animal models, among non-athletes as well as physically active individuals [56,57]. However, studies with dietary supplements containing polyphenols have provided inconsistent results [58,59].

Martarelli and Pompei (2009) noticed individual differences in the response to oxidative stress due to dietary habits among 40-year-old athletes participating in a 24 h mountain bike race [60]. The positive influence of total dietary antioxidant intake on the training capacity and recovery in males was also observed in the study of Devrim-Lanpir concerning ultra-endurance athletes [61]. Among fruit-derived polyphenols, pomegranate and tart cherry supplementation appeared to be beneficial in terms of antioxidant and anti-inflammatory effects as well as exercise performance and post-exercise recovery [62,63].

**Figure 1:**
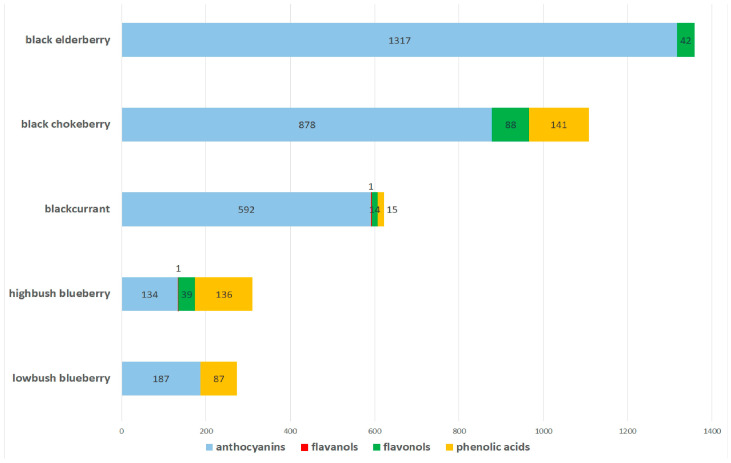
Polyphenol content in the reviewed berries [mg/100 g fresh fruit]. Prepared on the basis of data contained in Phenol-Explorer database.

As berry fruits are at the very top of the list of the richest polyphenol food sources, supplements containing berries have become a subject of interest in the context of counteracting exercise-induced oxidative stress. Particular attention was paid to the berries containing the highest amounts of polyphenols, i.e., chokeberries, black elderberries, blueberries, and blackcurrants [28]. A comparison of the content of particular kinds of polyphenols in the aforementioned berries is presented in Figure 1.

The findings of studies assessing the effects of supplements including the above berries in response to exercise are included in Table 1.

Based on the available literature, blackcurrant supplementation, with its richest version being New Zealand blackcurrant (NZBC) extract, is the most commonly explored berry fruit. The effects of NZBC supplementation were assessed by Lyall et al. (2009) on a group of 10 healthy individuals completing 30 min of rowing/day for 3 weeks [64]. The authors observed improved antioxidant and anti-inflammatory responses during and post-exercise in the intervention group. Over 20% higher lipid oxidation was noticed in the group of trained cycling females and experienced ultra-endurance runner in both cases after 7 days of supplementation [66,77]. Lipid oxidation has previously been considered to enhance exercise capacity among 34 healthy male volunteers participating in the Copenhagen Ironman Competition [88]. The period of 7-day supplementation was also found to be effective in improving repeated cycling performance (dose 300 mg/day), climbing time and endurance (600 mg/day), as well as muscle oxygenation [65,70,71]. Costello et al. (2020) examined 9 days of blackcurrant supplementation (7 days prior + 2 days after) with a dose of 300 mg twice a day among half-marathon runners. However, no significant difference was found in muscle soreness and post-exercise muscle damage recovery in comparison to the placebo group [69]. These findings are in line with the results of some other authors [68,89]. Paton et al. (2022), in the study of 12 experienced male cyclists, did not note any beneficial effects of a single application of a blackcurrant extract [89]. Similarly, the single use of 200 mL of NZBC juice before the treadmill test in the study of 40 healthy volunteers revealed no significant effect on blood lactate, glucose, and plasma MDA levels in comparison to the placebo group [68]. In contrast to the above observations, Hunt et al. (2021) documented beneficial effects of NZBC among 27 healthy volunteers undertaking a strenuous resistance trial [74]. Supplementation of 3 g per day for 12 days (8 days prior and 4 days after the exercise test) resulted in faster post-exercise muscle recovery, reduced muscle soreness, and a lower serum CK concentration.

The influence of chokeberry supplementation on exercise-induced oxidative stress was the subject of only a few studies. Pilaczynska-Szczesniak et al. (2005) observed the antioxidative effect of chokeberry juice supplementation (150 mL/day for 1 month) in the study of rowing training camp participants both during physical exercise and recovery [79]. Similar beneficial effects of longer chokeberry supplementation (7–12 weeks) were obtained among endurance trained adults [80,82,83]. Interestingly, the results of the single use of 200 mL of chokeberry juice before a half-marathon race suggest that it could be a solution against elevated platelet reactivity associated with prolonged running [81].

Supplementation with blueberries was found to be beneficial in several studies including physically active individuals. The study performed by McAnulty et al. (2011) revealed that supplementation of 250 g of blueberries per day for 6 weeks and 375 g 1 h prior to 2.5 h of running resulted in a reduction in the inflammation process and oxidative stress [84]. In contrast, the randomized controlled trial performed among recreational runners showed that 4 days of blueberry powder supplementation did not influence exercise performance [86]. However, this intervention lowered the blood lactate response to running as compared to the placebo group.

Even though black elderberry is one of the richest source of polyphenols among berries, we have not found any studies assessing the influence of black elderberry supplements on exercise-induced biomarkers and exercise performance so far.

## 4. Supplementation of Selected Berry-Derived Polyphenols and Cardiovascular Response

The phenomenon of oxidative stress also affects the cardiovascular system. Excessive ROS production (such as that produced during intense physical exercise) can cause endothelial dysfunction, generate inflammation, activate platelets, increase lipid oxidation, and consequently, can accelerate the progression of atherosclerosis [90,91,92].

Dark fruits, like the aforementioned berries, are rich sources of anthocyanins that have already been proven to enhance cardiovascular health and prevent the deterioration of endothelial function [93,94,95,96]. Their importance even seems to have increased in the reality of COVID-19 [97]. The beneficial effect of berry-derived polyphenols in reducing systolic and diastolic blood pressure and in the positive modification of blood lipids was observed not only in primary prevention but also among patients with cardiovascular and metabolic diseases [98,99,100,101,102].

As reports on the effects of polyphenols on cardio-metabolic biomarkers increase, research on their impact on the cardiorespiratory response, especially in trained individ-226 uals, still remains limited. Importantly, in the available literature, studies on the effect of chokeberry supplementation on cardiovascular parameters in physically active individuals were not found. Furthermore, out of seven relevant studies, two experiments were performed with the use of blueberry and the rest with blackcurrant supplementation. Park et al. (2018) observed an increased maximal oxygen uptake (VO_2max_) in a group of eight active young men who consumed blueberry extract. However, no significant differences in heart rate (HR), glucose, and blood lipid profile were noted [85]. Brandenburg and Giles (2021) also found no effect of 4 days of blueberry powder supplementation on blood oxygen saturation during exercise as well as the average and maximum HR during a 30 min treadmill test among 11 experienced runners [86].

As for blackcurrant supplementation, Pastellidou et al. (2021) did not observe a substantial influence on time to exhaustion at or above the critical speed, and on VO_2max_ in a study of 15 male runners [76]. These findings are in line with the majority of studies presenting no significant CVD effects with blackcurrant supplements regardless of the number of doses or the duration of the supplementation. In the study involving twelve trained cyclists, the administration of a single dose of a blackcurrant-extract beverage provided no beneficial effect on cycling performance or physiological measures relative to the placebo group [89]. While acute supplementation seems insufficient, longer-term supplementation may result in CVD changes. Cook et al. (2023) assessed the effects of NZBC extract on the isometric-contraction-induced cardiovascular responses. During 7 days of supplementation (600 mg per day), measurable effects were observed only after a few days of the experiment. The authors suggested that the bioavailability of blackcurrant anthocyanins and anthocyanin-derived metabolites was required for days to alter the mechanisms for CVD responses [78]. However, these findings are inconsistent with the results obtained by Montanari et al. (2021), who showed that NZBC extract administered for a week was not effective in enhancing cardiovascular function during rest and submaximal exercise in endurance-trained cyclists [75]. Interestingly, Willems et al. (2018) indicate that the ergogenic responses to anthocyanin intake from NZBC might be ethnicity-dependent [67].

## 5. Summary and Conclusions

Numerous studies have documented that high-intensity or prolonged exercise is associated with increased oxidative stress and modification of antioxidant status. However, this relationship is complex and depends on a number of factors including the age of the athlete, type of exercises and training, the sport season, etc. Interestingly, the reactive species produced during a bout of exercise may be beneficial, resulting in specific training adaptations like angiogenesis, mitochondria biogenesis, and muscle hypertrophy [37,103,104]. On the other hand, intensive exercises may provoke the excessive generation of free radicals, leading to damage of skeletal muscle fibers and fatigue. In this context, polyphenol-rich dietary supplements seem to be compounds that can upregulate the endogenous antioxidant defense system and consequently prevent muscle damage and support recovery.

Therefore dietary antioxidant supplements, including fruit-derived polyphenols, have been studied among highly active individuals as a potential method to prevent exercise-induced oxidative stress [105,106]. Of note, the majority of previous reviews included studies assessing the effects of mixtures of antioxidants, e.g., powders or beverages containing polyphenols and vitamins or other compounds. The present review focuses on the findings of experiments with the use of berry-derived polyphenols alone in order to more directly explore their properties.

Although several experiments showed beneficial effects of berry-derived supplements with polyphenols, some discrepancies remain. Due to inconsistencies in methodologies, direct comparison of the obtained results is limited. As presented, a wide range of doses were used (300 mg–24 g/day) with different frequencies/durations (from a single use to 12 weeks) and forms (juice, fresh fruit, powder) of supplementation with berry-derived polyphenols. The interval between supplementation and training also varies considerably from 1 h before to even several days after the trial. Divergent results may also depend on the fact that anthocyanins, one of the main types of polyphenols in dark fruits, have rather low bioavailability in comparison with other flavonoids due to their degradation in the gastrointestinal tract, mainly caused by the intestinal microbiota [107,108].

Importantly, of all the analyzed berries, most of the evidence has been gathered regarding the NZBC extract properties. Therefore, the conclusions concerning supplements containing NZBC seem to be the strongest.

Leaving aside the numerous inaccuracies and gaps in research results, due to their high polyphenol content, berry-derived supplements present a promising element in the prevention of exercise-induced oxidative stress and CVD outcomes.

As official recommendations for the use of polyphenol supplements are not justified at this moment, further research in this area is required. It is also important for safety reasons, because athletes often reach for dietary supplements even if evidence on their bioavailability and effectiveness is not based on unequivocal findings.

There is a list of unanswered issues, like the type of supplements, particular polyphenol sources, dosages, etc., that can pose exciting challenges for researchers. As most studies exploring the antioxidant properties of polyphenols were performed in vitro, on animal models, or among sedentary individuals, there is a need to gather more evidence on the effects of berry-derived supplements from studies including physically active individuals and professional athletes. Apart from NZBC extracts, other berry-derived supplements should also be explored more thoroughly. Due to the high polyphenol content in black elderberry (the second richest source of polyphenols after chokeberries), it would be interesting to know the effects of black-elderberry-derived supplementation on the antioxidant status of athletes. Of note, raw elderberries are poisonous for humans, but when heat-processed, they are entirely safe [28,109].

In summary, it should be emphasized that the use of berry-derived supplements seems to be an interesting method of regulating the oxidative status and cardiovascular protection in physically active individuals. Due to several unanswered questions and gaps in the evidence, further in-depth research seems necessary in this field.

However, regardless of any supplementation, well-balanced nutrition rich in natural antioxidants should remain a priority, not only for athletes, but for society as a whole.

## Figures and Tables

**Table 1:** Selected studies on the influence of berry-fruit-derived supplementation on exercise-induced changes in physically active individuals.

First Author and Publication Year	Supplementation Details	Research Group	Main Results
Blackcurrant			
Lyall 2009 [64]	120 mg/d of NZBC extract before and 120 mg/d after trial of 30 min rowing at 80% VO_2max_ for 3 weeks with a week washout	10 healthy physically active participants (5 males and 5 females); age = 48 ± 2.5 yrs	Lower CK activity generated by exercise was 30% lower immediately after test and 40% lower 24 h post-exercise in comparison to placebo group, suppressed exercise-induced oxidative stress (plasma carbonyls 30% decreased in intervention group)
Murphy 2017 [65]	300 mg/d of NZBC extract for 7 days prior to 2 × 4 km cycling separated by 10 min of active recovery	10 experienced male cyclists; age = 30 ± 12 yrs	0.82% faster total time and 7 Watts higher mean power of cycling trial; no effect on lactate level and HR during both parts of the trial and recovery
Strauss 2018 [66]	600 mg/day NZBC extract for 7 days before the trial of 120 min cycling at 65% VO_2max_	16 endurance-trained females; age = 28 ± 8 yrs	Lipid oxidation increased by 27%, 12% lower mean rate of carbohydrate oxidation, pre-exercise non-esterified fatty acids elevated by 49% and glycerol concentrations in response to NZBR extract supplementation increased by 27%; no effect on HR and % VO_2max_
Willems 2018 [67]	600 mg/d NZBC extract for 7 days before performed 30 min treadmill walk at 5 MET	17 healthy Thai men; age = 22 ± 3 yrs	Both stroke volume and cardiac output increased by 12%, systemic vascular resistance decreased by 10%
Lomiwes 2019 [68]	200 mL of NZBC drink containing total polyphenol concentration of 4.8 mg/kg bodyweight 1 h before treadmill exercise (walking for up to 2 h)	40 healthy volunteers (15 males and 25 females); aged 20 and 59 years old	90% decline in platelet MAO-B activity, 30% decrease in post-exercise lactate response, increase in self-motivation to walk (11 min longer time and over 10 km distance achieved by 20% more of participants); no effect on blood glucose level or HR
Costello 2020 [69]	600 mg/d of NZBC extract for 7 days prior and 2 days after half marathon	20 experienced recreational runners (8 females and 12 males); age = 30 ± 6 yrs	No effect on perceived muscle soreness and post-exercise muscle recovery
Fryer 2020 [70]	600 mg/day NZBC extract for 7 days before the test consisting of the 40% maximal volitional contraction	12 intermediate male rock climbers; Age = 26 ± 5 yrs	Improved forearm muscle oxygenation (min TSI% significantly lower, during 3 min recovery TTHR of TSI% significantly faster); no difference in time to exhaustion
Potter 2020 [71]	600 mg/day NZBC extract for 7 days before the trial involving 3 self-paced climbs with 20 min recovery after each climb	18 intermediate male sport climbers; age = 24 ± 6 yrs	Lower rating of perceived exertion in the intervention group, improved maintenance of total climbing time (TCT) and climbing endurance (post-exercise lactate levels gradually decreased in subsequent trials). No effect on mean HR, peak HR during the trial, and HR during recovery; right handgrip strength higher in placebo group
Willems 2020 [72]	600 mg/day NZBC extract for 7 days before the trial of sixteen, 5 s voluntary maximal isometric contractions separated by 3-s rest	12 recreationally active males; age = 24 ± 5 yrs	Higher total force production of 9–12% in the first stage of the trial
Fryer 2021 [73]	600 mg/day NZBC extract for 7 days before the trial of two submaximal forearm muscle contractions at 60% of their maximal volitional contraction with 5 min of passive recovery between	12 advanced male rock climbers; age = 25 ± 4 yrs	Higher muscle oxidative capacity (37% reduction in O_2_HTR), lower min-TSI% during the two exhaustive exercise in intervention group. No effect on time to exhaustion
Hunt 2021 [74]	3 g/day NZBC extract for 8 days prior to and 4 days following exercise test involving 60 strenuous concentric and eccentric contractions of the biceps brachii muscle on an isokinetic dynamometer	27 healthy non-resistance trained volunteers (19 females and 8 males); age = 23.5 ± 2 yrs)	Faster post-exercise muscle recovery (reduction in maximal voluntary contraction observed within first 24 h), reduced muscle soreness, serum CK concentration increased significantly after the trial in placebo remaining unchanged in intervention group
Montanari 2021 [75]	Two doses: 300 mg and 600 mg/day of NZBC extract for 1 week of submaximal cycling (65% VO_2max_) on day 1 (D1), D4, and D7	13 male trained cyclists; age = 39 ± 10 yrs	Intake of 600 mg for 7 days increased by 2.5% stroke volume and cardiac output, and lowered total peripheral resistance by 6.5%
Pastellidou 2021 [76]	300 mg/d NZBC extract for 3 days before high-intensity treadmill running	15 recreationally active males; age = 24.4 ± 3.6 yrs	No effect on time to exhaustion at or above critical speed, VO_2max_, lactate threshold, submaximal running economy or substrate utilization during exercise
Willems 2022 [77]	600 mg/d NZBC extract for 7 days before trial of 2 h treadmill running (speed: 10.5 km/h, 58% VO_2max_)	1 experienced ultra-endurance male runner; age = 40 yrs	The respiratory exchange ratio was lower by 00.2 units, and carbohydrate oxidation by 11%; fat oxidation was 23% higher
Cook 2023 [78]	600 mg/d NZBC extract supplementation for 1, 4, and 7-days while performing 120 s submaximal (30%) isometric contraction of the knee extensors	19 male participants; age = 26 ± 4 yrs	Larger femoral artery, mean arterial pressure, stroke volume, cardiac output were changed at time points during the isometric contraction following 7 days of intake in comparison to 1 day of intake of NZBC extract. No changes observed in SBP and DBP, HR, and total peripheral resistance
Chokeberries			
Pilaczyńska-Szczęśniak 2005 [79]	150 mL of chokeberry juice daily (3 doses of 50 mL) for 4 weeks; 2000 m rowing exercise test with workload increased every 3 min from 40–90% of maximal power at the beginning and at the end of suppl. period	19 male professional rowers; age = 21 ± 0.8 yrs	Lower lactate levels after exercise test, reduced TBARS concentrations 1 min after the exercise test and following a 24 h recovery period, GPx activity lower 1 min after the exercise test, SOD activity lower after 24 h recovery, lower CK activity 1 min after the test
Cikiriz 2020 [80]	30 mL/d of chokeberry extract for 12 weeks	16 male handball players during competition season with everyday regular training; age = 20.26 ± 2.86 yrs	Decreased levels of prooxidants (TBARS and nitrites), reduced glutathione levels and increased catalase activity, and level of high-density lipoprotein No changes in VO_2max_
Stevanović 2020 [81]	200 mL of chokeberry juice before a simulated half-marathon race	10 healthy male recreational runners; age = 30.8 ± 2.3 yrs	Decrease in the expression of platelet activation markers No effect on platelet aggregation
Stankiewicz 2021 [82]	200 mL (2 × 100 mL) of chokeberry juice/day, for 7 weeks Before and after supplementation period, the maximal multistage 20 m shuttle run test (the “beep test”) was performed	20 male semi-professional football players participating in regular for game season physical exercise program; age = 15.8 ± 0.7 yrs	No effect on VO_2max_, antioxidant capacity (TBARS, 8-OHdG, lactate), blood morphology, nor inflammation parameters
Vidović 2022 [83]	100 mL chokeberry juice/day for 8 weeks of participation in 60 min moderate-to-high-intensity aerobics classes 3 times a week	28 healthy women performing less than 30 min of intense or 60 min of moderate activity per week in preceding 3 months; age = 25.1 ± 2.8 yrs	Decrease in total cholesterol, GPx, SOD, and catalase activity
Blueberries			
McAnulty 2011 [84]	250 g/d of fresh blueberries for 6 weeks and 375 g 1 h prior to 2.5 h of running at ~72% maximal oxygen consumption	25 well-trained runners; age = 32.3 ± 14.3 yrs	NK cell counts 96%, 122%, and 76% greater, respectively, 1 h before, immediately after, and 1 h after trial; reduced oxidative stress (74% lower F2-isoprostanes post-exercise increase) and increased anti-inflammatory cytokines (IL-10 almost 100% higher than in control group).
Park 2018 [85]	7-day intake of 140 g frozen pulp extract of fresh blueberries before VO_2max_ test on a treadmill	8 active non-smoking male runners; age = 23 yrs	Increased exercise performance time and VO_2max_; significant decrease in IL-6, CRP, glucose, and insulin levels. No changes in ratings of perceived exertion, HR, and lipid profile
Brandenburg 2019 [86]	24 g/d packet of freeze–dried blueberry powder supplementation for 4 days prior to an 8 km time trial	14 recreational runners; age = 31.3 ± 10.3 yrs	Over 20% reduced post-exercise lactate response. No influence on HR, rating of perceived exertion, and running performance
Brandenburg 2021 [87]	24 g/d packet of freeze-dried blueberry powder supplementation for 4 days prior to two sessions of a 30 min treadmill trial	11 experienced runners (4 males; 7 females); age = 28.4 ± 7.5 yrs	Lower blood lactate response (20% post-exercise decrease in comparison to control group). No differences in fraction of exhaled nitric oxide (FENO), distance achieved, HR, oxygen saturation, and rating of perceived exertion

## Data Availability

No new data were created.
